# Supplementary material for: Proteasome-dependent degradation and nucleus–vacuole junctions sustain proteostasis during acute glucose starvation
Source: bioRxiv. 2026 May 12:2026.04.22.720209. Originally published 2026 Apr 25. Preprint. [Version 2] doi: 10.64898/2026.04.22.720209 (PMC13131599; doi:10.64898/2026.04.22.720209)
Supplement: 1 [file NIHPP2026.04.22.720209v2-supplement-1.pdf]

bioRxiv preprint doi: https://doi.org/10.64898/2026.04.22.720209; this version posted May 12, 2026. The copyright holder for this preprint (which was not certified by peer review) is the author/funder, who has granted bioRxiv a license to display the preprint in perpetuity. It is made available under aCC-BY 4.0 International license.

Proteasome-dependent degradation and nucleus–vacuole junctions sustain proteostasis during acute glucose starvation

Mihaela Pravica[1], Dina Franić[1], Matko Bazdan[1], Dominik Guzalić[1], Antonio Bedalov[2], Mirta Boban[1*]

Supporting information

- **Supplementary figures S1 – S3**

bioRxiv preprint doi: https://doi.org/10.64898/2026.04.22.720209; this version posted May 12, 2026. The copyright holder for this preprint (which was not certified by peer review) is the author/funder, who has granted bioRxiv a license to display the preprint in perpetuity. It is made available under aCC-BY 4.0 International license.

Supplementary Figure S1

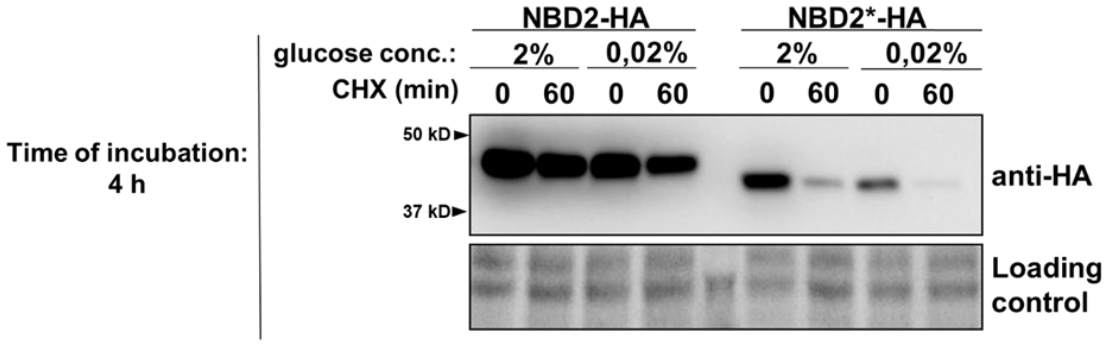

Supplementary figure S1. Selective degradation of the misfolded protein NBD2*-HA is maintained during acute glucose starvation. Representative immunoblots of native NBD2-HA and the misfolded variant NBD2*-HA. Exponentially growing yeast cells expressing NBD2-HA or NBD2*-HA were subjected to acute glucose depletion (0.02% glucose) or glucose replete conditions (2% glucose) as a control, for 4 h. Protein stability was assessed by cycloheximide chase assay (CHX, 100 µg/mL) by analyzing protein levels at indicated time points by Western blot (anti-HA). Stain-free total protein is shown as a loading control.

bioRxiv preprint doi: https://doi.org/10.64898/2026.04.22.720209; this version posted May 12, 2026. The copyright holder for this preprint (which was not certified by peer review) is the author/funder, who has granted bioRxiv a license to display the preprint in perpetuity. It is made available under aCC-BY 4.0 International license.

Supplementary Figure S2

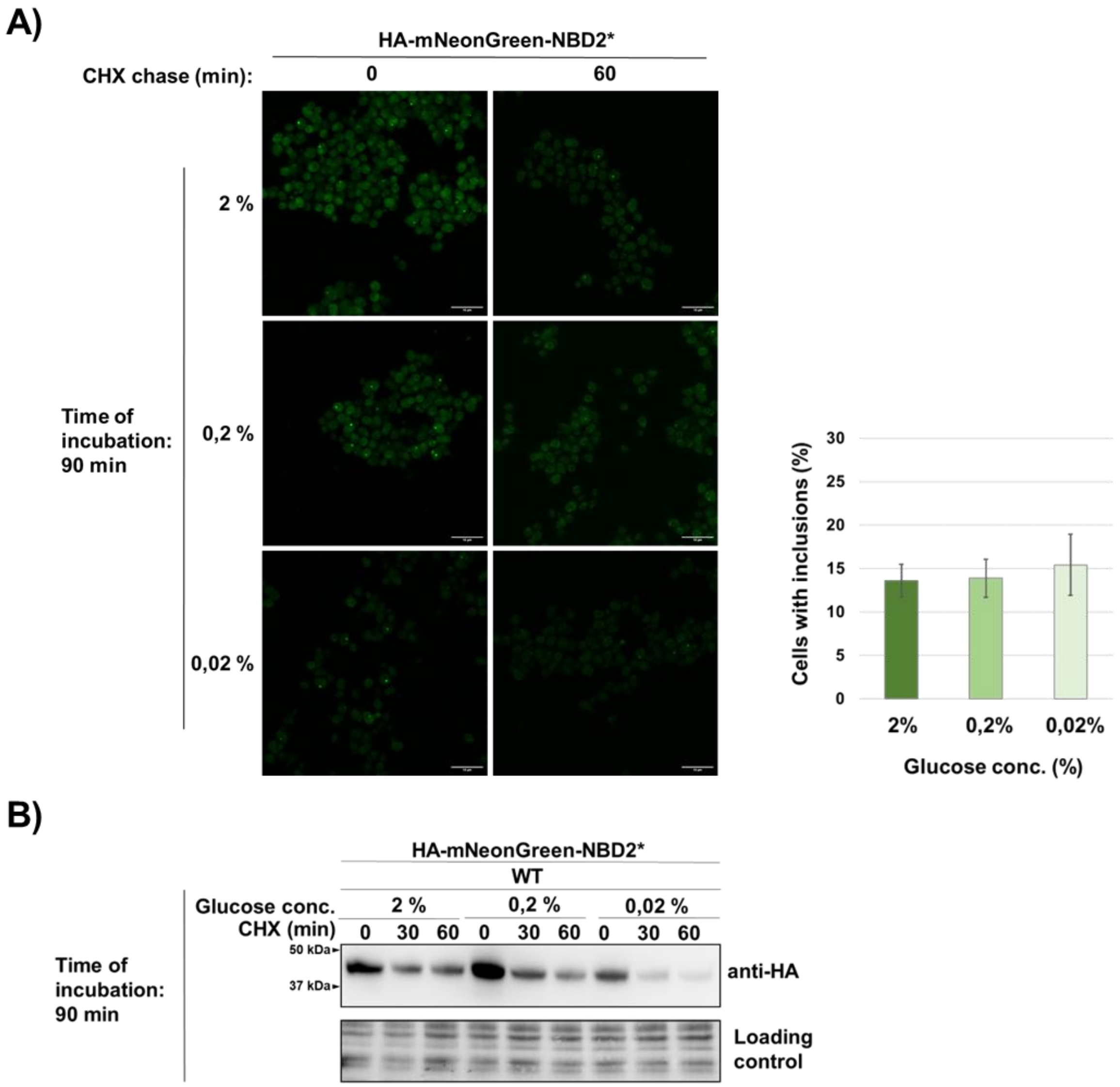

Supplementary figure S2. Selective degradation of HA-mNeonGreen-NBD2* persists during acute glucose starvation in *S. cerevisiae*. A, Representative images of yeast cells expressing HA-mNeonGreen-NBD2* under glucose-replete conditions (media containing 2% glucose) or acute glucose starvation (media containing 0.02% glucose) for 90 min. Yeast cells expressing HA-mNeonGreen-NBD2* were exponentially grown in the medium containing 2% glucose, and either maintained under glucose replete conditions (2% glucose) or subjected to acute glucose depletion (0.02% glucose) for 90 min prior to imaging by confocal microscopy. Images show whole Z-stacks. Scale bar, 10 µm. The graph shows the percentage of cells that contain inclusions. Data are expressed as mean ± SD from two independent experiments. **B,** Representative immunoblots showing turnover of the model misfolded protein HA-mNeonGreen-NBD2*. Cells grown as in (A) were subjected to cycloheximide chase assay (CHX, 100 µg/mL), harvested at indicated time points after cycloheximide addition and protein levels were analyzed by Western blot (anti-HA). Stain-free total protein is shown as a loading control.

bioRxiv preprint doi: https://doi.org/10.64898/2026.04.22.720209; this version posted May 12, 2026. The copyright holder for this preprint (which was not certified by peer review) is the author/funder, who has granted bioRxiv a license to display the preprint in perpetuity. It is made available under aCC-BY 4.0 International license.

Supplementary Figure S3

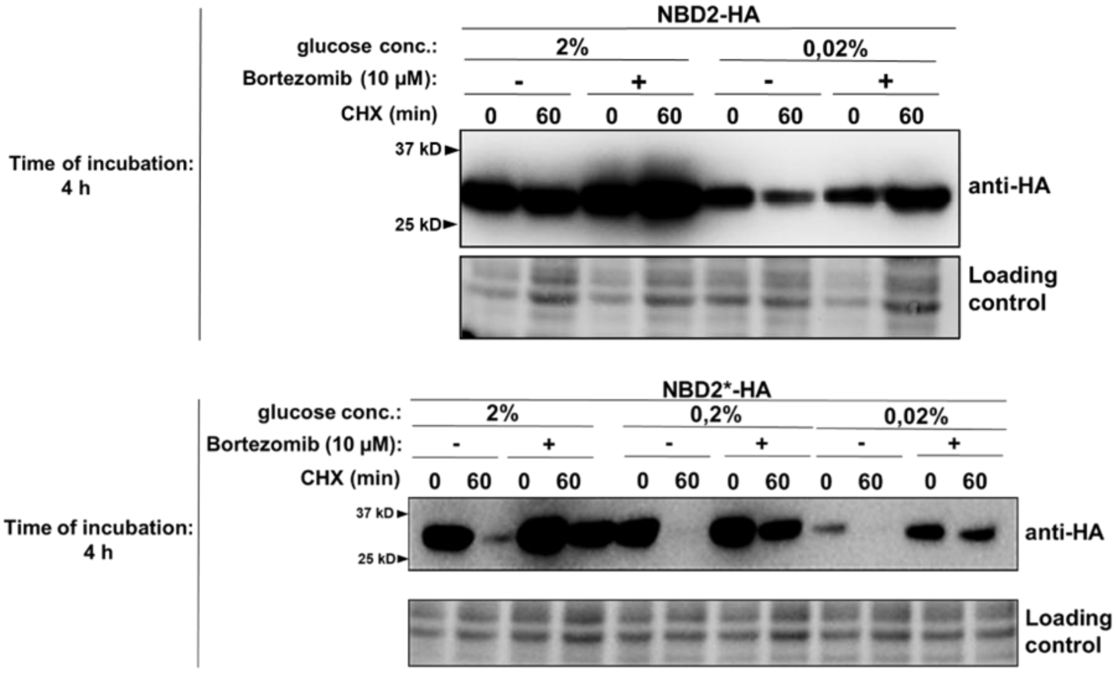

Supplementary figure S3. Degradation of NBD2*-HA during acute glucose starvation depends on proteasomal proteolytic activity. Representative immunoblots of NBD2-HA and NBD2*-HA in the presence or absence of proteasomal inhibition. Exponentially growing yeast cells expressing NBD2-HA or NBD2*-HA were subjected to acute glucose depletion (0.02% glucose) or glucose replete conditions (2% glucose) as a control, for 4 h. Protein stability was assessed by cycloheximide chase assay (CHX, 100 µg/mL) by analyzing protein levels at indicated time points by Western blot (anti-HA). Proteasome dependency was evaluated by treating cells with proteasomal inhibitor bortezomib (10 µM) for 30 min before cycloheximide addition. Stain-free total protein is shown as a loading control.
