# Supplementary material for: Proteasome-dependent degradation and nucleus–vacuole junctions sustain proteostasis during acute glucose starvation
Source: bioRxiv. 2026 Apr 25:2026.04.22.720209. Preprint. [Version 1] doi: 10.64898/2026.04.22.720209 (PMC13131599; doi:10.64898/2026.04.22.720209)

bioRxiv preprint doi: https://doi.org/10.64898/2026.04.22.720209; this version posted April 25, 2026. The copyright holder for this preprint (which was not certified by peer review) is the author/funder, who has granted bioRxiv a license to display the preprint in perpetuity. It is made available under aCC-BY 4.0 International license.

SUPPORTING INFORMATION

SUPPLEMENTARY FIGURES

Figure S1. Misfolded NBD2*-HA is targeted for degradation during the late phase of acute glucose starvation.

Representative immunoblots characterizing the turnover of native NBD2-HA and the misfolded variant NBD2*-HA. Logarithmically growing *S. cerevisiae* cells were shifted to either glucose-replete medium (2% glucose) or subjected to prolonged acute starvation (0.02% glucose) for 4 h. Following the starvation period, protein stability was assessed by cycloheximide chase assay (100 μg/mL) over the indicated time course to monitor the degradation of pre-existing protein pools. Total protein loading was visualized and normalized using Stain-Free technology.

Figure S2. Selective degradation of HA-mNG-NBD2* persists during acute glucose starvation in *S. cerevisiae*.

A) Representative confocal fluorescence microscopy images of log-phase yeast cells expressing HA-mNG-NBD2* under glucose-rich YPD (2%) or glucose-deprived (0.02%) conditions for 90 min. Images represent maximum intensity projections of Z-stacks. Scale bar, 10 μm. The bar chart depicts the proportion of cells in the culture that contain inclusions. Data are expressed as mean ± SD from two independent experiments.

B) Representative immunoblots showing turnover of the model misfolded protein HA-mNG-NBD2*. Log-phase yeast cells were subjected to 90 min acute glucose starvation (0.02% glucose) or control 2% glucose YPD medium, followed by cycloheximide chase assay (100 μg/mL) at the indicated time points. Total protein loading was visualized using Stain-Free technology.

Figure S3. NBD2*-HA continues to undergo proteasome-mediated degradation during acute glucose depletion

Representative immunoblots showing the degradation of NBD2-HA and NBD2*-HA in the presence or absence of proteasomal inhibition. Log-phase yeast cells expressing NBD2-HA and NBD2*-HA were subjected to acute glucose starvation (0.02% glucose) for or 4 h (late stage of glucose depletion) prior to a cycloheximide (CHX) chase assay (100 μg/mL) to monitor protein turnover. Proteasome dependency was evaluated by pretreating cells with 10 μM bortezomib for 30 min before CHX addition. Total protein loading was visualized using Stain-Free technology.

bioRxiv preprint doi: https://doi.org/10.64898/2026.04.22.720209; this version posted April 25, 2026. The copyright holder for this preprint (which was not certified by peer review) is the author/funder, who has granted bioRxiv a license to display the preprint in perpetuity. It is made available under aCC-BY 4.0 International license.

Figure S1

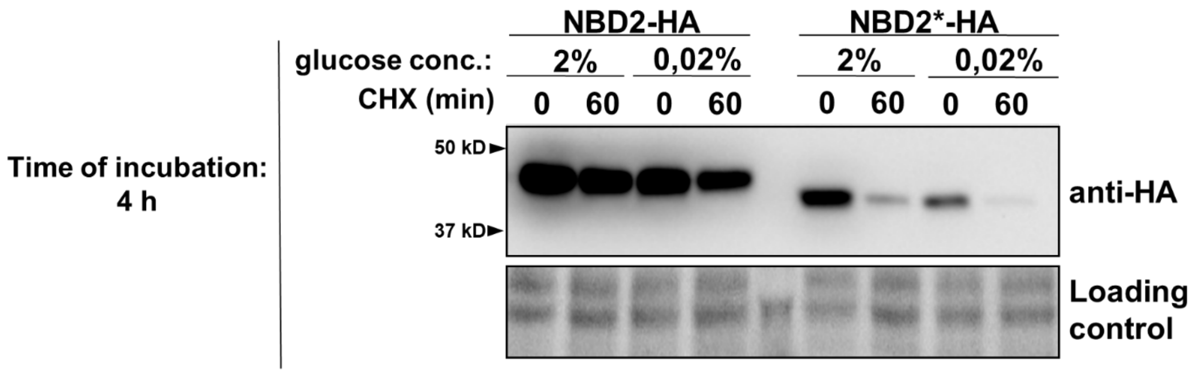

bioRxiv preprint doi: https://doi.org/10.64898/2026.04.22.720209; this version posted April 25, 2026. The copyright holder for this preprint (which was not certified by peer review) is the author/funder, who has granted bioRxiv a license to display the preprint in perpetuity. It is made available under aCC-BY 4.0 International license.

Figure S2

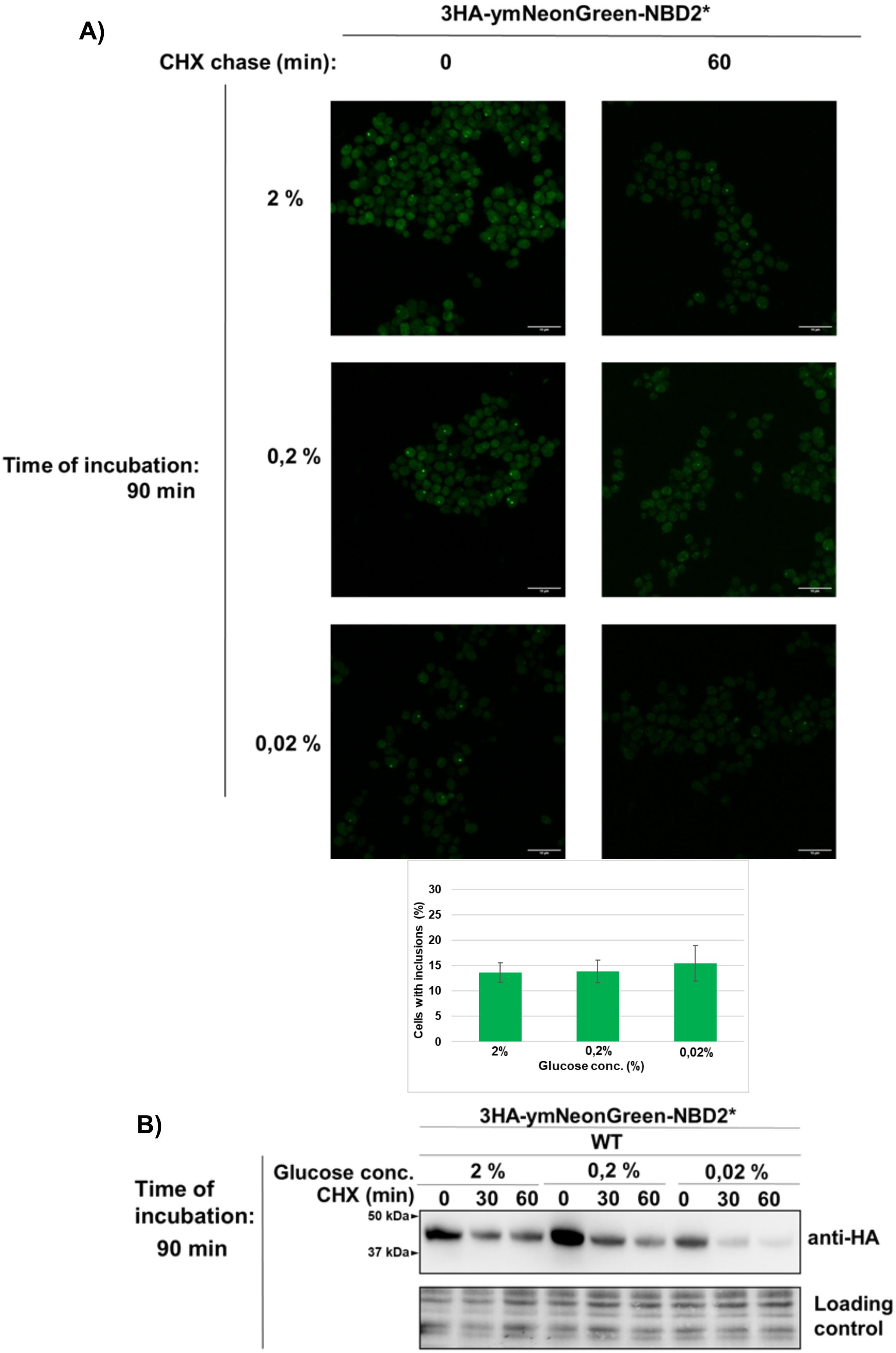

bioRxiv preprint doi: https://doi.org/10.64898/2026.04.22.720209; this version posted April 25, 2026. The copyright holder for this preprint (which was not certified by peer review) is the author/funder, who has granted bioRxiv a license to display the preprint in perpetuity. It is made available under aCC-BY 4.0 International license.

Figure S3

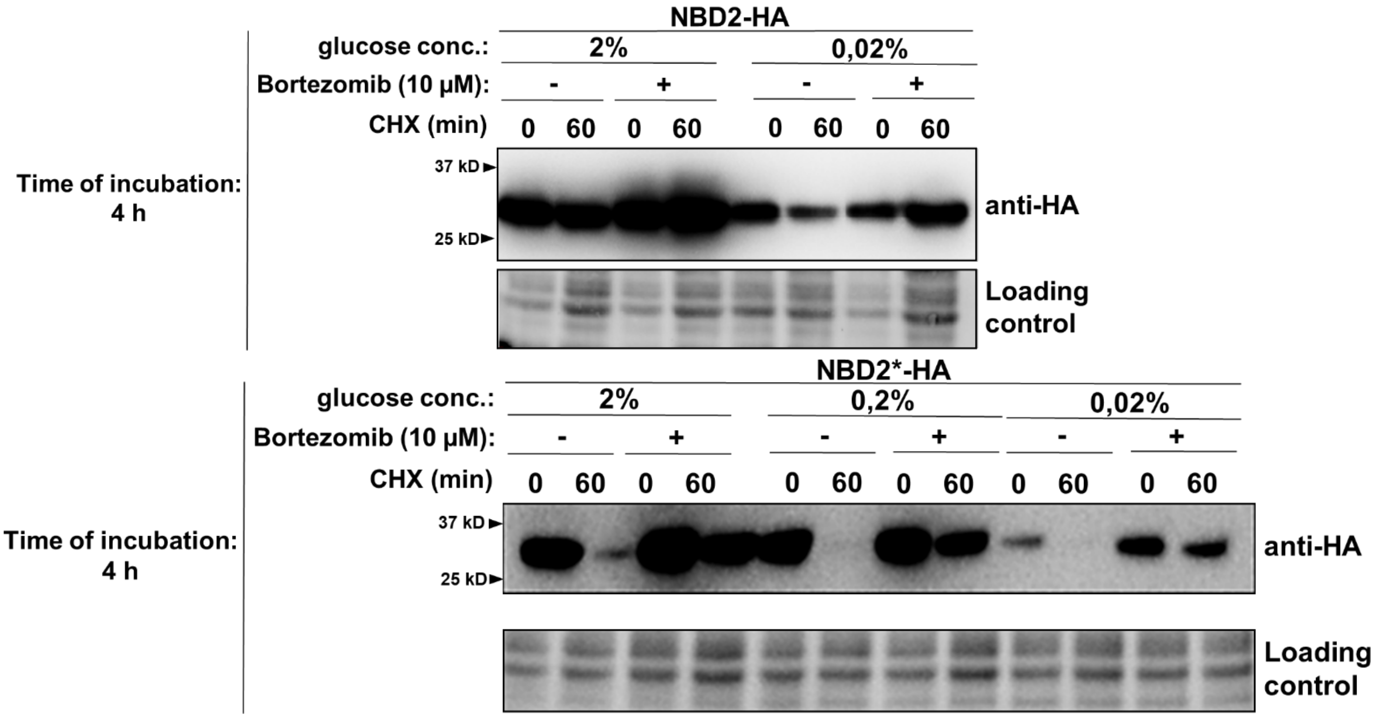

Supplement: Supplementary file 1 [file NIHPP2026.04.22.720209v1-supplement-1.pdf]
